# Men’s Mental Health Matters: The Impact of Traditional Masculinity Norms on Men’s Willingness to Seek Mental Health Support; a Systematic Review of Literature

**DOI:** 10.1177/15579883251321670

**Published:** 2025-05-27

**Authors:** Leshata Winter Mokhwelepa, Gsakani Olivia Sumbane

**Affiliations:** 1 School of Medicine, Faculty of Health Sciences, University of Limpopo, Polokwane, South Africa

**Keywords:** traditional masculinity norms, men’s mental health, help-seeking behavior, barriers to mental health care

## Abstract

Men’s mental health is a focus area for improving population health worldwide. Traditional masculine norms among men have been reported to make them more susceptible to mental health issues. This study aims to review existing literature on how traditional masculinity norms influence men’s willingness to seek mental health support. This review followed stages of systematic review. The review search was initiated in February 2024. The search process was conducted on electronic databases such as PubMed, PsycINFO, Science Direct, and Google Scholar to identify relevant studies published in English between 2000 and 2024. The search terms included “traditional masculinity norms,” “men’s mental health,” “help-seeking behavior,” and “barriers to mental health care.” Studies were selected based on their relevance to the topic. The inclusion criteria were studies focusing on adult men and examining the relationship between traditional masculinity norms and mental health help-seeking behaviors. Exclusion criteria included studies focusing on children, adolescents, or women and nonempirical articles. Data extraction covered study details, design, population characteristics, and key findings. This study included 47 relevant studies. Two key themes emerged, namely: masculinity and mental health (impact on mental health); and barriers to seeking help. Traditional masculinity norms significantly deter men from seeking mental health support, highlighting the need for culturally sensitive interventions that address these barriers.

## Introduction

There is substantial evidence that men are reluctant to seek mental health treatment across diverse global contexts, spanning both high- and low-income settings (Staiger et al., 2020). Particularly, men’s mental health experiences are shaped by deeply ingrained social norms surrounding masculinity, which also affect men’s behavior, attitude, and mode of expression (Gough & Novikova, 2020). Men’s views against accessing mental health services are becoming more negative due to widespread gender role-related issues, such as conventional masculinity’s connection to mental illness and perceptions that seeking help undermines one’s masculinity (Pederson & Vogel, 2007). The term “masculine” refers to a category of ideas, emotions, and behaviors that are typically associated with men and often perpetuated through societal expectations, media portrayals, and peer influences (Kim & Yu, 2023; Levant & Pryor, 2020).

Research frequently indicates that enduring conventional masculine norms, such as being strong, successful, independent, in charge, and capable, along with an emphasis on avoiding emotions, is a key contributor to men’s aversion to help-seeking behavior when it comes to depression (Möller-Leimkühler, 2009). Depression is characterized as being “incompatible” with traditional masculinity because it is associated with feminine emotional experiences, frequently results in feelings of helplessness and loss of control, and often leaves sufferers feeling weak and vulnerable (Emslie et al., 2006). The impact of rigid masculine norms on male roles in relation to men’s attitudes toward depression and help-seeking was corroborated by the results of a systematic analysis of qualitative studies on men’s perspectives of depression (Krumm et al., 2017). According to research involving 13,884 Australian men, men’s risk of attempting suicide increased dramatically when they adhered to masculine standards of emotional suppression and stoicism (Pirkis et al., 2017). The negative impact of adhering to traditional masculine norms on how men experience depression and seek treatment is further substantiated by a systematic review of the function of masculinity in scenarios where depressed men participate in help-seeking behavior (Seidler et al., 2016). Studies have shown that these patterns are not unique to specific regions but are found across different cultures, though the severity and expression of these norms may vary. In South Africa, for example, mental health issues contributed to 13,774 deaths in 2019, of which 10,861 were men (Ngwenya & Sumbane, 2022).

In the United States, men are expected to be stoic, self-sufficient, and in control according to traditional male values imposed by the dominant culture (Mahalik et al., 2003). These traits are typically incompatible with asking for assistance. Although the definition of “traditionally masculine” varies depending on the social and cultural setting, the underlying theme of emotional suppression as a marker of strength appears consistent across different cultural contexts (Liu, 2005). According to academics, all males in the United States need to “come to terms with the concepts of masculinity that the prevailing society has” (Mahalik et al., 2003). Boys are therefore inundated with messages such as “boys don’t cry” from a young age (Vogel et al., 2011). Boys and men who early learn that others would not react favorably to them are less likely to display mental health symptoms to others as a result of these signals. This social conditioning creates a significant barrier to emotional openness, even when men are suffering from severe psychological distress. For example, after seeing a film that shows an emotion-focused counseling session, males who experience more gender role conflict have fewer positive opinions of counseling and psychotherapy and are less likely to indicate a willingness to seek counseling (Rochlen et al., 2004).

Strong adherence to hegemonic masculine norms puts men in “double jeopardy,” as evidenced by increased psychological anguish and decreased readiness to ask for assistance (Levant et al., 2011). Men may so frequently endure in silence due to the societal belief that emotional vulnerability equates to weakness (Levant et al., 2011). One potential explanation for how males initially come to adhere to masculine norms has been suggested: gendered social learning (Levant et al., 2011). According to this theory, males learn to conform to masculine norms through a long history of social punishment and reinforcement in a range of social settings. Men may be more or less ready to adhere to masculine norms depending on the particular social situations, because this type of learning is context specific (M. Addis, 2011). Men might, for example, be more eager to show emotional vulnerability when they are with their spouse because they have previously been rewarded for this behavior, but they might not be as willing to do so when they are with their male friends since they may have previously been punished for this behavior (M. Addis, 2011). Getting help for mental health problems is a class of actions that can be appropriate in some situations but inappropriate in others (M. Addis, 2011). Most of the earlier research on men’s help-seeking behavior has been focused on self-report measures and noncontextual research, which limits our understanding of the complexity of help-seeking behaviors in different social and cultural environments (Whorley & Addis, 2006).

Men are experiencing more emotional suffering as a result of the global COVID-19 pandemic, which has exacerbated underlying issues related to mental health, isolation, and emotional well-being (Ogrodniczuk et al., 2021). Previous research indicates that during the COVID-19 epidemic, adult men experienced higher stress levels, increased isolation, and worsening mental health (Ogrodniczuk et al., 2021). It is interesting to note that during this pandemic, there has been a growing tendency in the number of men seeking emotional help, indicating a potential shift in help-seeking behaviors when faced with unprecedented global crises (Ellison et al., 2021).

Given these complexities, the goal of this review is to examine the body of research on the influence of conventional masculinity norms on men’s willingness to seek mental health treatment. Our review aims to provide a comprehensive understanding of the factors contributing to men’s reluctance to seek help and the implications for mental health services. This research has significant implications for policy development and the creation of more effective interventions that consider the role of gender norms in mental health care.

## Materials and Method

### Study Design

A systematic review and meta-synthesis make up the study design. We chose to use a theme synthesis in this review, as suggested by Thomas and Harden’s (2008) recommendation. To create descriptive themes, pertinent material from the included studies’ results sections is extracted, coded inductively and line-by-line, and then grouped into related categories. The framework for this review was provided by the PRISMA (Preferred Reporting Items for Systematic Reviews and Meta-Analysis) declaration (Moher et al., 2015). A systematic review is needed to synthesize fragmented and inconsistent research on traditional masculinity norms and men’s mental health help-seeking behaviors. It fills a gap by providing a comprehensive overview, identifying knowledge gaps, and offering evidence-based insights for targeted interventions. This helps clarify the broader impact of masculinity norms and informs future research and policy. Therefore, the research question posed was: What is the impact of conventional masculine norms on men’s inclination to pursue mental health assistance?

### Study Selection

**Figure 1.**
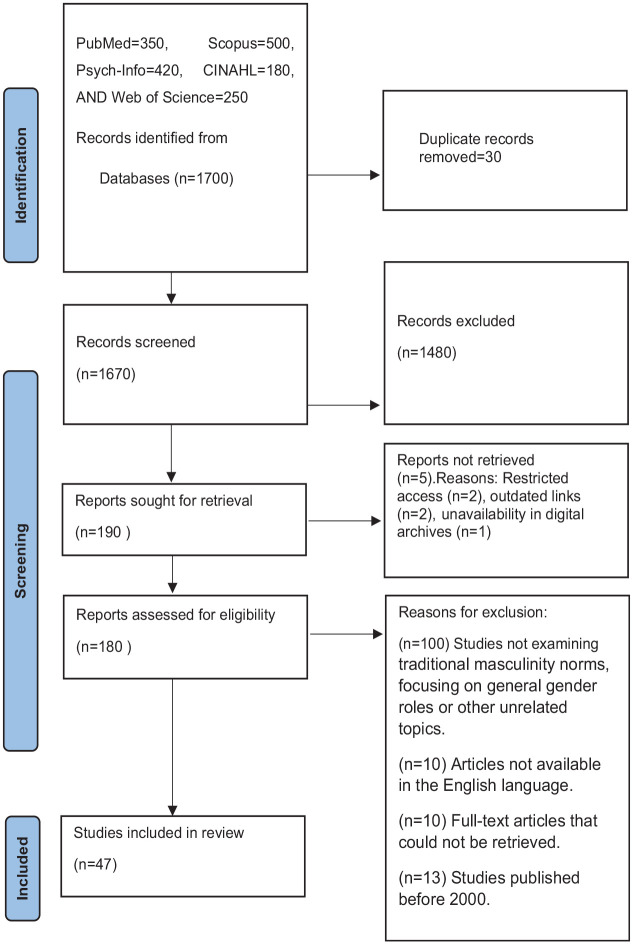
A Flowchart Diagram Representing the Search Strategy (Agrawal et al., 2024)

We used Population, Exposure, and Outcome (PEO) as eligibility criteria which is shown in Table 1 (Moola et al., 2015). PEO was chosen because it is specifically designed for qualitative and observational studies, which are the primary focus of this systematic review. The topic of traditional masculinity norms and their impact on men’s mental health does not involve interventions but rather explores associations, behaviors, and experiences within a defined population. A thorough search of English written literature was conducted in January 2024. The search was conducted on electronic databases such as PubMed, PsycINFO, CINAHL, Scopus, and Web of Science. A full search strategy is as follows: PubMed [370] (“men’s mental health” [MeSH] OR “mental health” [MeSH] OR “male mental health”) AND (“traditional masculinity norms” OR “masculinity” [MeSH] OR “masculinities” OR “gender norms” [MeSH]) AND (“help-seeking behavior” [MeSH] OR “mental health services” [MeSH] OR “mental healthcare utilization”); PsycINFO [430] ((“men’s mental health” [MeSH] OR “mental health” [MeSH] OR “male mental health”) AND (“traditional masculinity norms” OR “masculinity” [MeSH] OR “masculinities” OR “gender norms” [MeSH]) AND (“help-seeking behavior” [MeSH] OR “mental health services” [MeSH] OR “mental healthcare utilization”)); CINAHL [200] (MH “Men’s Health” OR MH “Mental Health Services”) AND (MH “Masculinity” OR MH “Masculinities” OR MH “Gender Identity”) AND (MH “Help-Seeking Behavior” OR MH “Mental Health Services”); Scopus [520] (TITLE-ABS-KEY (“men’s mental health” OR “male mental health” OR “mental healthcare utilization”) AND TITLE-ABS-KEY (“traditional masculinity norms” OR “masculinity” OR “masculinities” OR “gender norms”) AND TITLE-ABS-KEY (“help-seeking behavior” OR “mental health services”)); Web of Science [270] TOPIC: (“men’s mental health” OR “male mental health” OR “mental healthcare utilization”) AND TOPIC: (“traditional masculinity norms” OR “masculinity” OR “masculinities” OR “gender norms”) AND TOPIC: (“help-seeking behavior” OR “mental health services”). The total number of 1,700 articles was searched. Five articles were not retrieved because of access restrictions. It is very important to note that the authors reviewed English-language studies published from 2000 to 2024 to ensure that the review encompasses relevant, recent, and methodologically sound research. This range captures the evolution of societal attitudes toward masculinity and mental health, providing a comprehensive overview of how traditional masculinity norms impact men’s willingness to seek mental health support. Therefore, the selection process for the included studies is shown in Figure 1.

**Table 1.** Summary of Eligibility Criteria

Criterion	Inclusion criteria	Exclusion criteria
Population/participants	• Adult men (18+), any geographical location, studies published in English	• Adolescents or children, studies focusing on women or nonbinary individuals without comparative data
Exposure	• Traditional masculinity norms related to emotional suppression, self-reliance, societal expectations	• Studies not examining traditional masculinity norms, focusing on general gender roles or other unrelated topics
Outcome	• Willingness to seek mental health support, including both formal and informal support	• Studies not measuring or discussing men’s willingness to seek mental health support

### Quality Appraisal

The Critical Appraisal Screening Program for Qualitative Studies was used to evaluate the methodological quality of the included studies (Long et al., 2020). The CASP tool was selected because the focus of our review required evaluating the depth and quality of studies that explore perceptions, behaviors, experiences, and concepts often addressed through qualitative methodologies. While the quantitative studies provided valuable data, our primary objective was to assess how traditional masculinity norms influence men’s mental health and willingness to seek support, which often requires a nuanced understanding of context and lived experience. The CASP tool is well suited for assessing the methodological rigor and trustworthiness of qualitative research, ensuring the quality of the studies included in this review (Long et al., 2020). Ten methodological domains are the focus of this tool, which is designed to assess their quality. Each methodological domain is represented by an item that has three possible scores: Yes (Y), No (N), and Uncertain (U), based on whether the item has been adequately described in the article’s full text. Higher scores denote a higher caliber of study. Based on the overall scores obtained, the authors of earlier studies established the quality ranges as low (0–5.5), medium (6–8.5), and high (9–10; Basso et al., 2024). The quality appraisal checklist questions are as follows: (1) Was there a clear statement of the aims of the research? (2) Is a qualitative/quantitative methodology appropriate? (3) Was the research design appropriate to address the aims of the research? (4) Was the recruitment strategy appropriate to meet the aims of the research? (5) Was the data collected in a way that addressed the research issue? (6) Was the relationship between researcher and participant adequately considered? (7) Were ethical issues taken into consideration? (8) Was the data analysis sufficiently rigorous? (9) Is there a clear statement of findings? (10) How valuable is the research?

All included studies obtained higher scores of 10, deeming high quality.

### Data Extraction

A standardized Microsoft Excel spreadsheet was utilized by two researchers (M.L.W. and S.G.O.) to extract data from specific experiments, as indicated in Table 2. To ensure high standards and reduce the possibility of bias, the authors additionally invited one reviewer for a comprehensive assessment of this review. The reviewer arbitrated any disagreement.

**Table 2.** Summary of Included Studies

Author, year	Study method	Country/location	Population (*n*)	Age range (years)	Gender of the participants	Outcomes/results
Wong et al. (2015)	Quantitative study	United States	183	18 or older	Males	Men with comparatively low self-esteem were most affected by masculinity priming in terms of self-esteem.
Levant et al. (2011)	Quantitative study	United States	323	18 or older	Males	The relationship between male gender socialization and health behavior varies depending on the particular masculine gender socialization construct and health behavior facet.
Kimmel & Mahalik (2004)	Quantitative study	United States	154	18 or older	Males	Higher degrees of body dissatisfaction and masculine norm compliance.
Kimmel & Mahalik (2005)	Quantitative study	United States	357	18 or older	Males	The adherence of homosexual males to masculine standards did not correlate with negative body image and high level of distress.
Magovcevic & Addis (2008)	Quantitative study	United States	102	18 or older	Males	Measures of depressive symptoms and adherence to masculine norms showed a moderate correlation with externalizing symptoms.
Rice et al. (2011)	Quantitative study	Australia	1,223	Older than 18	Males	Masculinity norms predisposed men to high levels of depression.
Levant & Wimer (2014)	Quantitative study	United States	585	Older than 18	Males	Social theory norms affected men’s mental health.
Liu & Iwamoto (2007)	Quantitative study	United States	154	18 or older	Males	Men reported to use substances and consume alcohol as a coping mechanism.
Rankin (2013)	Quantitative study	Germany	77	Above 18	Males	High mental health levels.
Wong et al. (2017)	Quantitative study	United States, Australia, Canada	78	Above 18	Males	Conforming to male standards was linked to poor social functioning more so than it was to psychological markers of poor mental health.
Oliffe et al. (2011)	Qualitative study	Canada	22	55–79	Males	Stigma was the cause of depression and suicidal thoughts in men.
Lynch et al. (2018)	Qualitative study	Europe	17	18–24	Males	Masculinity norms were found to be the barrier to access mental health services.
Emslie et al. (2006)	Qualitative study	United Kingdom	16	Above 18	Males	High level of depression.
McKenzie et al. (2018)	Qualitative study	New Zealand	15	20–40	Males	There is a strong correlation between masculinity norms and mental health.
Gulliver et al. (2012)	Qualitative study	Australia	15	16–23	Males	The biggest perceived obstacle to young elite athletes seeking aid was stigma.
Lysova et al. (2022)	Qualitative study	Australia, Canada, United Kingdom, United States	41	28–63	Males	The majority of participants who refrained from seeking assistance did so because of their own incapacity to identify abuse and gauge their own risk of harm, as well as attempts to maintain family unity, macho stereotypes, and justifications for their partner’s abuse.
Courtenay (2010)	Quantitative study	United States	50	Above 18	Males	Poor mental health seeking help.
Ramaeker & Petrie (2019)	Quantitative study	United States	423	18 or older	Males	Risk-taking and other masculine norms associated with higher alcohol consumption and gender role conflicts, such as those between work and family, significantly predicted depressive symptomatology. In addition, self-stigma partially mediated the relationship between conforming to masculine norms and negative attitudes about and intentions to seek psychological help across both groups of men.
Barragán (2024)	Qualitative study	United States	7	At least 18	Males	Men face several obstacles to receiving mental health care.
Clary et al. (2023)	Qualitative study	United States	26	18 or older	Males	Therapy could help more than half of people who are struggling with mental health issues and who do not seek care.
Mostoller & Mickelson (2024)	Quantitative study	United States	326	18–75	Males	Support of masculinity standards was strongly correlated with reported stress, but not with depression, in people who sought therapy due to self-stigma.
Doornbos et al. (2024)	Qualitative study	United States	50	23–83	Males	Men were found to encounter mental problems due to stigma.
Scott (2018)	Mixed method study	Canada	6	At least 18	Males	The participants’ help-seeking behaviors were most significantly impacted by the ideology of traditional masculinity in that they aligned with several traditional masculinity/help-seeking.
Teese et al. (2023)	Quantitative study	Australia	2,170	18–29	Males	Numerous results-specific conclusions were also shown outside of the norms that are hegemonically male.
Peralta (2007)	Qualitative study	United States	78	18–24	Males	According to the findings, drinking in public is seen as a way to demonstrate one’s masculinity.
Farrimond (2012)	Qualitative study	Europe	14	18 or older	Males	Many interviewees used the myth of the “Neanderthal Man,” who shuns physicians, to justify men’s typical tendency to seek treatment.
Boysen & Logan (2017)	Quantitative study	United States	984	18 or older	Males	Increased stigma was associated with masculine illnesses, while gender atypicality had no discernible impact.
Boysen (2017)	Quantitative study	United States	634	18 or older	Males	Fear was a stigmatizing attitude that was exclusively connected to men and masculinity.
Courtenay (2000)	Qualitative study	United States	50	18 or older	Males	Men typically utilize these harmful social practices as tools to negotiate social power and status and as markers of their masculinity.
Vogel & Heath (2016)	Quantitative study	United States	50	Above 18	Males	Men seek mental health less than women.
Sileo & Kershaw (2020)	Quantitative study	United States	119	18–25	Males	At baseline, more substance use was correlated with male toughness.
Burns & Mahalik (2011)	Qualitative study	United States	15	At least 18	Males	Masculinity norms triggered suicidal thoughts among males.
O’brien et al. (2005)	Qualitative study	Scotland and Asia	55	17–72	Males	Men negotiate deviations from the hegemonic view of help-seeking.
Berger et al. (2005)	Quantitative study	United States	155	18–88	Males	The findings show that men with higher scores on tests of gender role conflict and conventional masculinity ideology also typically have more unfavorable attitudes regarding seeking out professional assistance.
De Visser & McDonnell (2013)	Mixed method	United States	731	Over 18	Males	Participants with more traditional gender role beliefs had more strict beliefs about the masculinity of various health behaviors.
Parent et al. (2018)	Quantitative study	United States	4,825	20–59	Males	Men were found to be depressed.
Emslie et al. (2006)	Qualitative study	United Kingdom	38	Over 18	Males	High levels of depression.
Hammond (2012)	Quantitative study	United States	674	18 and older	Males	The findings show that men with higher scores on tests of gender role conflict and conventional masculinity ideology also typically have more unfavorable attitudes regarding seeking out professional assistance.
Addis & Mahalik (2003)	Qualitative study	United States	10	18 or older	Males	There is no denying that many guys dealing with a variety of life challenges underuse traditional support systems.
Goodwill et al. (2020)	Mixed method	United States	273	18–30	Males	Results of the study indicate that treatments for depression should be customized to include elements of masculinity that are particularly important to young Black males.
Walther et al. (2023)	Quantitative study	Germany	144	25–50	Males	Conventional masculinity and unstable notions of manhood.
Bryde Christensen et al. (2023)	Quantitative study	Denmark	8	25–54	Males	Men were found to be emotionally restricted.
Chen & Dognin (2017)	Qualitative study	United States	3	26–78	Males	The veterans’ interactions with their family, health care providers, and psychotherapists may be impacted by hegemonic masculine ideals.
Vickery (2022)	Qualitative study	United Kingdom	19	29–74	Males	Mutual regard and a sense of shared knowledge of events in a group environment.
Schlichthorst et al. (2019)	Qualitative study	Australia	17	18 or older	Males	Some people believed that sharing content regarding mental health and suicide put their relationships with others and their reputation at danger.
Robertson et al. (2018)	Qualitative study	United Kingdom, United States, New Zealand, Canada, Australia, Scotland, Ireland, Europe	13	18 or older	Males	Appropriate partnerships were also seen as a necessary requirement for success and as crucial for maximizing intervention impact.
Griffith et al. (2012)	Qualitative study	United States	22	18 and older	Males	There were complex relationships between masculinity and health that differed depending on the construct and health outcome.

### Characteristics of Included Studies

The majority of the studies are quantitative (*n* = 23), qualitative (*n* = 21), and mixed method (*n* = 3), respectively. The United States was found to be most country with research on the relationship between traditional masculinity and men. US was the country with most research on traditional masculinity norms among men. Most studies focused on males who were over 18 years. The summary of the characteristics of included studies is shown in Table 2.

### Results

This study included 47 relevant articles. Two main themes emerged: masculinity and mental health (impact on mental health); and barriers to seeking help.

## Theme 1: Masculinity and Mental Health (Impact on Mental Health)

Traditional masculinity norms were consistently linked to a wide range of negative mental health outcomes among men. Six of the articles highlighted those societal expectations of men as emotionally stoic and self-reliant lead to significant emotional suppression, which exacerbates mental health issues such as anxiety and depression (Kimmel & Mahalik, 2004; Kimmel & Mahalik, 2005; Levant et al., 2011; Magovcevic & Addis, 2008; Rice et al., 2011; Wong et al., 2015). For example, published articles emphasized that men who conformed to these norms often reported higher levels of stress and depression due to the internalized pressure to remain strong and resilient, even in the face of emotional challenges (Levant & Wimer, 2014; Liu & Iwamoto, 2007; Oliffe et al., 2011; Rankin, 2013; Wong et al., 2017).

This emotional suppression was identified as a major barrier to seeking help for mental health issues. This study revealed that many men avoided seeking professional mental health services due to feelings of embarrassment or fear of being perceived as weak (Emslie et al., 2006; Gulliver et al., 2012; Lynch et al., 2018; Lysova et al., 2022; McKenzie et al., 2018; Oliffe et al., 2020; Wiggins, 2024). This reluctance was commonly tied to their deeply ingrained masculine identity (Courtenay, 2010). In Ramaeker and Petrie’s (2019) study, participants echoed this sentiment, reporting that admitting to vulnerability conflicted with their perception of masculinity, which further exacerbated their social isolation and psychological distress. This inability to seek support from others, whether friends, family members, or mental health professionals, created a vicious cycle where men’s mental health issues remained unaddressed and often worsened over time.

Three articles revealed a direct link between traditional masculinity norms and engagement in risky behaviors as a coping mechanism for unaddressed mental health issues (Barragán, 2024; Clary et al., 2023; Mostoller & Mickelson, 2024). For instance, participants engaged in behaviors such as binge drinking and substance use as a way of managing feelings of anxiety, depression, and stress (Doornbos et al., 2024). These behaviors, driven by societal pressure to maintain emotional stoicism, were also associated with negative physical and psychological outcomes. Similarly, Scott’s (2018) study demonstrated that men turned to substance abuse as a means of avoiding emotional expression, leading to a cycle of destructive behaviors that further deteriorated their mental health. Few articles elaborated on the long-term impacts of such behaviors, showing that binge drinking and drug use contributed not only to worsening mental health but also to increased morbidity and mortality rates among men (Peralta, 2007; Teese et al., 2023). These risky behaviors, which were often exacerbated by societal expectations of masculinity, ultimately led to higher incidences of chronic health conditions and premature death.

Farrimond’s (2012) study focused on the reluctance of men to utilize mental health services, a trend that is deeply rooted in masculine ideals of self-reliance. In Boysen and Logan’s (2017) study, participants consistently reported avoiding professional help due to fears of being judged or stigmatized for their perceived weakness. This avoidance perpetuated a cycle of untreated mental health issues, as men preferred to “tough it out” rather than seek assistance. A study done by Courtenay (2000) offered further insights into this phenomenon, revealing that even when men acknowledged their need for support, societal pressures discouraged them from taking action (Vogel & Heath, 2016). Participants described feelings of hopelessness, knowing they needed help but feeling unable to pursue it due to the cultural and societal barriers imposed by traditional masculinity norms (Burns & Mahalik, 2011; Sileo & Kershaw, 2020). This contributed to an increase in feelings of isolation, making it even more difficult for men to escape the cycle of declining mental health.

## Theme 2: Barriers to Seeking Help

In the 47 articles included in this review, several key barriers to seeking help for mental health issues among men were identified. One recurring theme was the fear of being judged as less masculine. Five of the articles reported that men expressed significant concerns about being perceived as weak or unmanly if they sought help for mental health problems (Barragán, 2024; Clary et al., 2023; Mostoller & Mickelson, 2024; O’brien et al., 2005; Peralta, 2007). For instance, O’brien et al. (2005) revealed that participants feared negative judgment from peers, family, and society at large, which often led them to suppress their mental health struggles rather than seek support. Similarly, one research demonstrated that men were concerned that seeking help would contradict societal expectations of them as strong, resilient, and self-reliant, which further reinforced their reluctance to pursue mental health services (De, Visser, & McDonnell, 2013).

A strong association between traditional masculinity norms and delays in seeking help was evident across seven of the articles (Berger et al., 2005). The study conducted by De Visser and McDonnell (2013) highlighted that men who adhered to masculine ideals, such as being emotionally stoic and self-reliant, were significantly more likely to delay or avoid seeking mental health services, even when experiencing severe distress. This reluctance was echoed in Parent et al.’s (2018) study where participants reported that they intentionally postponed seeking help because they believed they should be able to handle their mental health issues on their own, without external assistance.

Participants described a preference for male doctors over female doctors when they did seek care, driven by the belief that male doctors would better understand their health concerns and embody the competence associated with traditional masculinity (Emslie et al., 2006). This preference sometimes led to delays in care, as men would wait for access to male health care providers rather than seeing available female providers. Researchers further suggested that this preference contributed to differential doctor–patient communication, which negatively impacted the quality of care these men received (Emslie et al., 2006). In addition, they explored how traditional masculinity norms influenced men’s attitudes toward depression (Hammond, 2012). These studies revealed that depression was often perceived as incompatible with masculinity because it involved emotional experiences, such as powerlessness and vulnerability, that were viewed as feminine (Gough et al., 2016). Participants in article by Gough et al. described how societal pressures to maintain their roles as providers and protectors made it difficult for them to seek help for depression, fearing that it would undermine their status within their families and communities (M. E. Addis & Mahalik, 2003). Men reported feeling that seeking help for depression could result in being ridiculed or marginalized by others, further reinforcing their reluctance to pursue treatment.

Finally, two articles noted that while traditional masculine norms often discouraged help-seeking, some men found strength in these same norms during their recovery processes (Griffith et al., 2012; Walther et al., 2023). The researchers reported that participants viewed their journey through mental health challenges as a personal struggle that allowed them to prove their resilience and emerge stronger, aligning with the idea of masculinity as heroic and powerful (Bryde Christensen et al., 2023; Chen & Dognin, 2017; Robertson et al., 2018; Schlichthorst et al., 2019; Vickery, 2022). This alternative form of masculinity, while still tied to traditional ideals, offered a pathway for men to seek help without feeling that they were betraying their sense of masculinity.

## Discussion

This study aimed to explore how traditional masculinity norms impact men’s willingness to seek mental health support and identify barriers to help-seeking. This systematic review was needed to address the fragmented and inconsistent research on the relationship between traditional masculinity norms and men’s mental health help-seeking behaviors. The review filled a key gap by consolidating findings across different contexts, offering a clearer and more comprehensive understanding of how these norms affect help-seeking patterns among men. What we now know is that traditional masculinity norms consistently pose a barrier to help-seeking across cultures, yet some aspects of these norms can also be protective under certain conditions. The findings reveal that adherence to traditional masculinity norms has a profound negative effect on men’s mental health, with significant implications for both psychological well-being and help-seeking behaviors. This is in line with study conducted by Cough et al. which discussed about traditional masculinity norms, which emphasize emotional suppression, self-reliance, and stoicism and often discourage men from expressing vulnerability or seeking support. These norms contribute to a culture where emotional expression is seen as a sign of weakness, leading men to internalize their struggles and avoid discussing their mental health concerns. This emotional suppression can exacerbate mental health issues such as anxiety and depression, as men are less likely to engage in healthy emotional processing or seek professional help (Magovcevic & Addis, 2008; Rice et al., 2011).

The study also highlights critical barriers to seeking help that are rooted in traditional masculinity norms. Fear of judgment and concerns about social perception significantly deter men from accessing mental health services. Men often perceive seeking help as a threat to their masculine identity, fearing that it will lead to stigmatization or undermine their self-reliance (Levant & Wimer, 2014; Liu & Iwamoto, 2007; Rankin, 2013). This fear is compounded by a societal expectation that men should handle their problems independently, which further discourages them from reaching out for support (Goodwill et al., 2020). These barriers create a substantial obstacle to effective mental health care, as men may avoid seeking help until their issues become more severe or unmanageable. This reluctance not only delays treatment but also increases the risk of more serious mental health outcomes (Levant et al., 2009).

It is important to acknowledge that some studies suggest that traditional masculine norms can serve as a positive resource. For example, a study by Mahalik et al. (2003) found that certain aspects of traditional masculinity, such as strong work ethic and perseverance, could be leveraged to promote resilience and coping strategies. Similarly, a study by Addis and Mahalik suggests that traditional masculine norms, when adapted to encourage emotional expression and seeking help in specific contexts, can contribute positively to mental health (Levant & Richmond, 2008). These studies indicate that aspects of masculinity can be reframed and utilized to support mental health rather than solely being seen as barriers.

The review’s findings predominantly reflect the negative impacts of traditional masculinity norms; however, recognizing the potential positive aspects can offer a more nuanced understanding of their role in mental health. While these norms are often criticized for discouraging emotional expression and promoting harmful self-reliance, acknowledging their positive aspects such as resilience, perseverance, and leadership can provide a more balanced view (Connell & Messerschmidt, 2005). For instance, traits like mental toughness and a strong work ethic, commonly associated with traditional masculinity, can enhance resilience and coping strategies in challenging situations (Mahalik et al., 2003). Integrating these strengths into therapeutic contexts can lead to more culturally relevant and effective interventions, respecting and utilizing men’s values rather than solely focusing on their limitations (Lukersmith et al., 2024). This balanced perspective allows for a comprehensive understanding that highlights how traditional masculinity norms can be both a barrier and a resource in supporting mental health and well-being (Tyler & Slater, 2018). Practical implications of these findings are crucial for developing effective mental health interventions and policies. To address the impact of traditional masculinity norms on mental health, it is essential to challenge and reframe societal expectations surrounding masculinity. Public health campaigns and mental health programs should focus on normalizing emotional expression and reducing the stigma associated with seeking help. For instance, the “Movember” campaign raises awareness about men’s health issues, including mental health, by encouraging open discussions and fundraising for related causes (Lukersmith et al., 2024). Similarly, the “Heads Together” campaign, supported by the Royal Foundation, aims to change the conversation around mental health and encourage people to seek support (Tyler & Slater, 2018). These campaigns demonstrate effective strategies in addressing mental health stigma and promoting emotional openness.

## Limitations

This study’s limitations include several factors that may impact the validity and comprehensiveness of its findings. The restriction to English-language publications could have excluded relevant research from non-English sources, potentially missing important cultural and contextual perspectives on masculinity norms and mental health. Publication bias may also skew results, as studies with significant findings are more likely to be published and included. The heterogeneity in study methodologies and definitions further complicates the synthesis of results, making it challenging to draw generalizable conclusions. Finally, the focus on research from 2000 to 2024 may not fully capture historical perspectives on masculinity norms, which could provide additional context.

## Conclusion

This study explored the impact of traditional masculinity norms on men’s willingness to seek mental health support and identified key barriers to help-seeking behavior. The findings reveal that adherence to traditional masculinity norms, which emphasize emotional stoicism, self-reliance, and toughness, significantly impacts men’s mental health. These norms contribute to emotional suppression and a heightened stigma around seeking help, leading men to underreport mental health issues and avoid professional support. Fear of judgment and societal perceptions of weakness further deter men from accessing mental health services, reinforcing a cycle of isolation and untreated mental health problems.

The study highlights the need for targeted interventions that challenge and reframe traditional masculinity norms. Public health campaigns and mental health programs should focus on normalizing emotional expression and reducing stigma to encourage men to seek help without fear of judgment. Creating supportive environments and tailoring mental health services to be more inclusive can also address the barriers identified. Building supportive social networks and community resources that validate men’s experiences and promote help-seeking behaviors is crucial for improving mental health outcomes.
